# Maternal weight and paediatric health use: mediating role of adverse birth outcomes: a retrospective cohort study

**DOI:** 10.1186/s12884-023-05744-w

**Published:** 2023-07-31

**Authors:** Lisa M. Currie, Hilary K. Brown, Beth K. Potter, Steven Hawken, Doug Coyle, Shi Wu Wen, Mark Walker, Laura Gaudet

**Affiliations:** 1 School of Epidemiology and Public Health, University of Ottawa, 600 Peter Morand Crescent, Ottawa, ON K1G 5Z3 Canada; 2 University of Toronto, Toronto, ON Canada; 3 Ottawa Hospital Research Institute, Ottawa, ON Canada; 4 Kingston Health Sciences Centre, Kingston, ON Canada; 5 Queen’s University, Kingston, ON Canada

**Keywords:** Perinatal epidemiology, Pregnancy, Health Services, Obesity, Pediatrics

## Abstract

**Background:**

Maternal pre-pregnancy body mass index (BMI) and gestational weight gain (GWG) above or below recommendations have been associated with increased paediatric health service utilization as well as increased risk of adverse birth outcomes, including small for gestational age (SGA) and preterm birth (PTB). SGA and PTB are associated with numerous adverse health outcomes in the child, including delayed growth, motor and cognitive impairment. Previous research has identified birth weight and gestational age on the causal pathway in the association between maternal pre-pregnancy BMI and child hospital admissions, there are no studies to date to quantify this relationship across other areas of health service utilization, nor the impact of gestational weight gain. This study aimed to assess if SGA or PTB partially explain the association between maternal weight and paediatric health service utilization.

**Methods:**

The study population consisted of all women who delivered a singleton, live infant in Ontario between 2012 and 2014, and was assembled from data contained in the provincial birth registry. Health service utilization over the first 24 months following birth was examined by linking data from the registry with other provincial health administrative databases housed at ICES. The mediating roles of PTB and SGA were assessed using the Baron-Kenny method and causal mediation analysis.

**Results:**

A total of 204,162 infants were included in the analysis of maternal pre-pregnancy BMI and 171,127 infants were included in the GWG analysis. The small magnitude of association between maternal BMI and paediatric health service utilization impacted our ability to estimate the indirect effect of maternal BMI through adverse birth outcomes (adjusted indirect effect = 0.00). 56.7% of the association between below recommended GWG and increased hospitalizations was attributed to PTB, while 6.8% of the association was attributed to SGA.

**Conclusion:**

Paediatric hospitalizations may be partially attributable to PTB and SGA in children born to mothers with below-recommended GWG. However, maternal weight also appears to be related to increased paediatric health service utilization independent of PTB and SGA.

**Supplementary Information:**

The online version contains supplementary material available at 10.1186/s12884-023-05744-w.

## Introduction

Elevated maternal pre-pregnancy body mass index (BMI) and gestational weight gain (GWG) both below and above the recommended range are associated with adverse birth outcomes, including small for gestational age (SGA) and preterm birth (PTB) [1, 2]. SGA and PTB are associated with numerous adverse health outcomes in the child, including delayed growth, motor and cognitive impairment [3–5]. In a previous study, we observed that maternal pre-pregnancy BMI was associated with increased health service utilization in the child in the first 24 months of life, with higher rates of health care use among children born to mothers considered overweight or obese, relative to those with normal pre-pregnancy BMI, in a dose-dependent manner [6]. Further, GWG outside the recommended range for BMI, particularly below recommended GWG, was associated with increased paediatric health service utilization, regardless of maternal pre-pregnancy BMI [7].

The association between maternal weight and paediatric health outcomes may be partly explained by adverse birth outcomes, such as SGA or PTB. Cameron et al. previously investigated the relationship between maternal pre-pregnancy BMI and child hospital admissions and noted that birth weight and gestational age were on the causal pathway of this relationship [1]. This study aimed to quantify the potential mediating role of adverse birth outcomes in the relationship between maternal weight and paediatric health service utilization in the first 24 months of life. We hypothesized any observed increase in paediatric health care utilization associated with pre-pregnancy overweight/obesity or with GWG outside the recommended range would be at least partially mediated by the increased risk of adverse birth outcomes in infants born to persons with below or above recommended pre-pregnancy BMI and GWG.

## Methods

### Study design, population, and data sources

This population-based retrospective cohort study expands upon work previously published by Currie et al. [6]. In summary, this study assessed pediatric health service utilization in the first 24 months of life for all live-born, singleton infants born in Ontario, Canada from April 1, 2012 to March 31, 2014 that are identified within the Better Outcomes Registry & Network (BORN) birth registry database (www.bornontario.ca). BORN is a high-quality, comprehensive registry with 100% coverage of all births in Ontario and demonstrated good agreement between the data elements and patient records [8].

All data used in this study were accessed via ICES, an independent, non-profit research institute whose legal status under Ontario’s health information privacy law allows it to collect and analyze health care and demographic data, without consent, for health system evaluation and improvement. (www.ices.on.ca). These datasets were linked using unique encoded identifiers and analyzed at ICES. The BORN birth registry has been linked deterministically at ICES via maternal and newborn encrypted Ontario Health Insurance Plan (OHIP) numbers and other individual identifiers. Once linked, an ICES Key Number (IKN) was appended to allow each infant’s record to be linked with other health administrative databases. Episodes of hospitalization were acquired from the Canadian Institute for Health Information Discharge Abstract Database (CIHI-DAD), which captures all hospitalizations recorded by Ontario institutions. OHIP billing records were used to quantify number of physician visits. Emergency department (ED) visits were captured from the CIHI National Ambulatory Care Reporting System (NACRS) database. The ICES Registered Persons Database (RPDB) provided information on death and neighbourhood income quintile. [6].

### Exposure variable

The BORN registry was used to obtain information pertaining to maternal pre-pregnancy BMI. Maternal height and weight measures, captured at the first prenatal visit either via self-report or clinical measurement, were used to derive pre-pregnancy BMI in the BORN database using maternal weight (kilograms) divided by height squared (metres 2) [6]. We categorized BMI according to World Health Organization (WHO) criteria [9], with underweight classified as BMI < 18.5 kg/m^2^, normal weight as 18.5–24.9 kg/m^2^, overweight as 25-29.9 kg/m^2^, and obese as ≥ 30 kg/m^2^. We excluded records with maternal BMI values deemed to be implausible (i.e., pre-pregnancy BMI < 10 kg/m^2^ or > 80 kg/m^2^) [10]. GWG was captured as a continuous measure within the BORN database based on maternal pre-pregnancy weight subtracted from maternal weight at delivery. We categorized GWG as below recommended (below recommended weight gain), recommended, or above recommended (above recommended weight gain), based on Health Canada recommendations (Table S1) [11].

### Outcome measures

The outcomes were measures of paediatric health service utilization, including rates of hospitalizations, ED visits, and physician visits [12] in children during the first 24 months following birth, assessed using data from the DAD, NACRS and OHIP databases, respectively. We derived the total number of hospitalization episodes (excluding the birth hospitalization) that occurred over the two-year follow-up period, which is inclusive of any hospitalization regardless of duration, but did not include day surgery visits [6]. For the total number of ED visits, we did not count those records in NACRS that were classified as non-urgent visits in the ED (e.g., children accessing a routine physician visit through the ED) or same day surgery visits. It is possible that a child would have multiple health service encounters for a single episode of care [13]. For physician visits, we considered all billings for the same patient on a given calendar day with the same physician to be part of a single visit since the OHIP database is organized by billing codes rather than discrete visits (i.e., there may be more than one billing associated with a single encounter between patient and physician). We also excluded physician visits associated only with lab billing codes from the total count of physician visits. [6].

### Covariates

Potential confounders of the relationship between maternal pre-pregnancy BMI/GWG and infant health service utilization [14] were identified *a priori* and included infant sex, maternal age, maternal smoking status, pre-existing maternal medical conditions (i.e., diabetes, hypertension), and neighbourhood income quintile. [6].

### Mediators

The following mediators were included in the analysis: gestational age < 37 weeks (PTB) and SGA (< 10th percentile), informed by previous research [1]. We excluded all records with missing gestational age or birth weight. An algorithm was applied to identify and exclude records with implausible gestational age/birth weight combinations based on population normative values, using cut offs of values greater or less than five standard deviations from this value [15].

### Statistical **analyses**

We first described baseline characteristics of mothers and children using frequency distributions. Rates of health service utilization during the first 24 months of life were calculated separately for each type of health service [6]. The total number of visits was divided by the total person-time of follow-up and reported as visits per 1000 person-years.

**Fig. 1:**
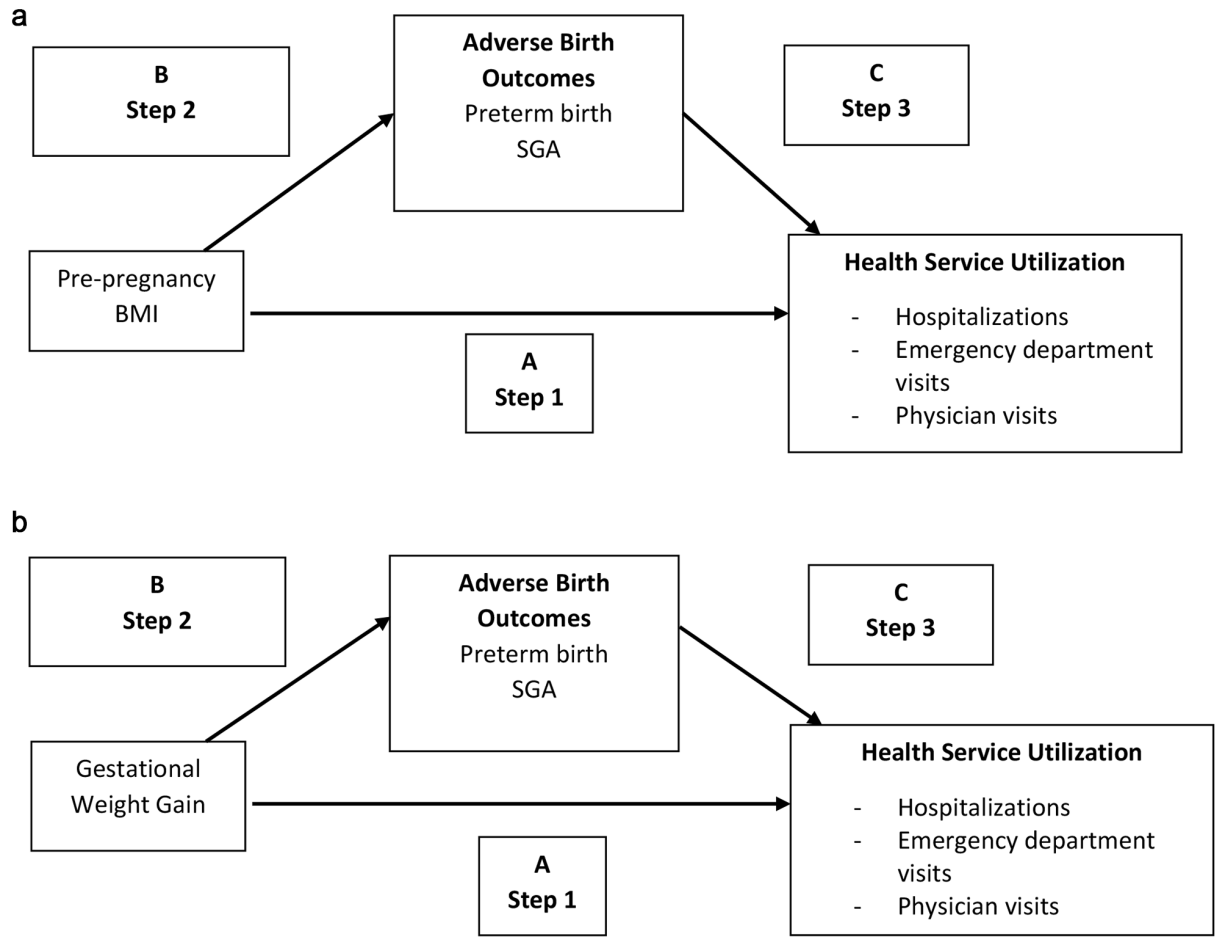
**a** Graphical representation of the mediation assessment of the relationship between pre-pregnancy BMI and paediatric health service utilization. **b**: Graphical representation of the mediation assessment of the relationship between gestational weight gain and paediatric health service utilization

To compare rates of infant health service utilization by maternal pre-pregnancy BMI and by GWG, we first reported unadjusted incidence rate ratios (IRRs) for health service utilization across BMI/GWG categories. We then used multivariable regression analysis to adjust for potential confounders. Due to over-dispersion, we used negative binomial regression [16] in two separate models (one for pre-pregnancy BMI and one for GWG) [8, 17]. A graphical representation of the mediation assessment of the relationship between the exposures and paediatric health service utilization is presented in Fig. 1.

### Mediation analysis

The Baron-Kenny approach was used to identify potential mediation and causal mediation analysis was used to investigate mediation when the Baron-Kenny approach indicated that it was present. The possible mediation of the relationship between maternal pre-pregnancy BMI or GWG and paediatric health service utilization, by PTB and small for gestational age [18], was assessed. This four-step approach included: step (1) Estimating the IRR for the exposure–outcome relationship (as described above); step (2) Estimating the IRR for the relationship of the exposure to the mediators; step (3) Estimating the IRR for the relationship of the mediators to the outcome; step (4) Estimating the IRRs for the relationship of the exposure and mediators to the outcome.

**Fig. 2:**
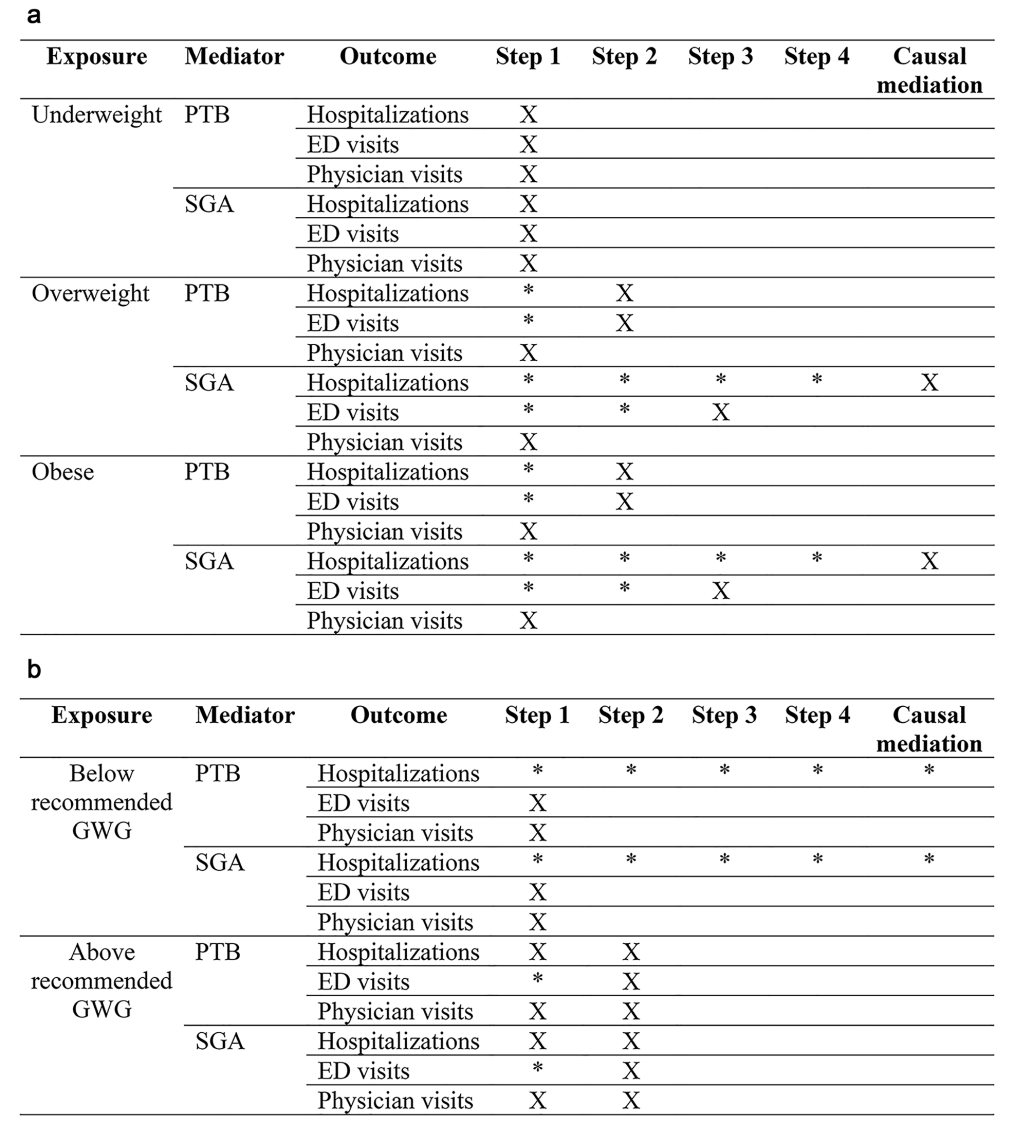
**a** Schematic of model construction for each stage of the Baron-Kenny method and causal mediation for maternal pre-pregnancy BMI (removed = X, significant = *). **b**: Schematic of model construction for each stage of the Baron-Kenny method and causal mediation for GWG (removed = X, significant = *)

In this mediation analysis approach, each step must demonstrate a significant relationship (either positive or negative) in order to proceed to the next step [18]. A conservative criterion of 0.90 > adjusted IRR [aIRR] < 1.10 was chosen for this analysis to identify results with effect sizes small enough to be considered non-meaningful. A relationship was removed from subsequent steps when it did not meet this criterion for mediation (Fig. 2a and b).

For mediation relationships that were apparent following the four steps described, SAS PROC CAUSALMED was used to ascertain the total, natural direct, and natural indirect effects to estimate the effect decomposition [19] and percent mediated for each relationship of maternal weight to outcome, addressing each mediator independently [20]. All analyses were completed using SAS Enterprise Guide 7.1 (SAS Institute, Cary, North Carolina).

## Results

**Fig. 3:**
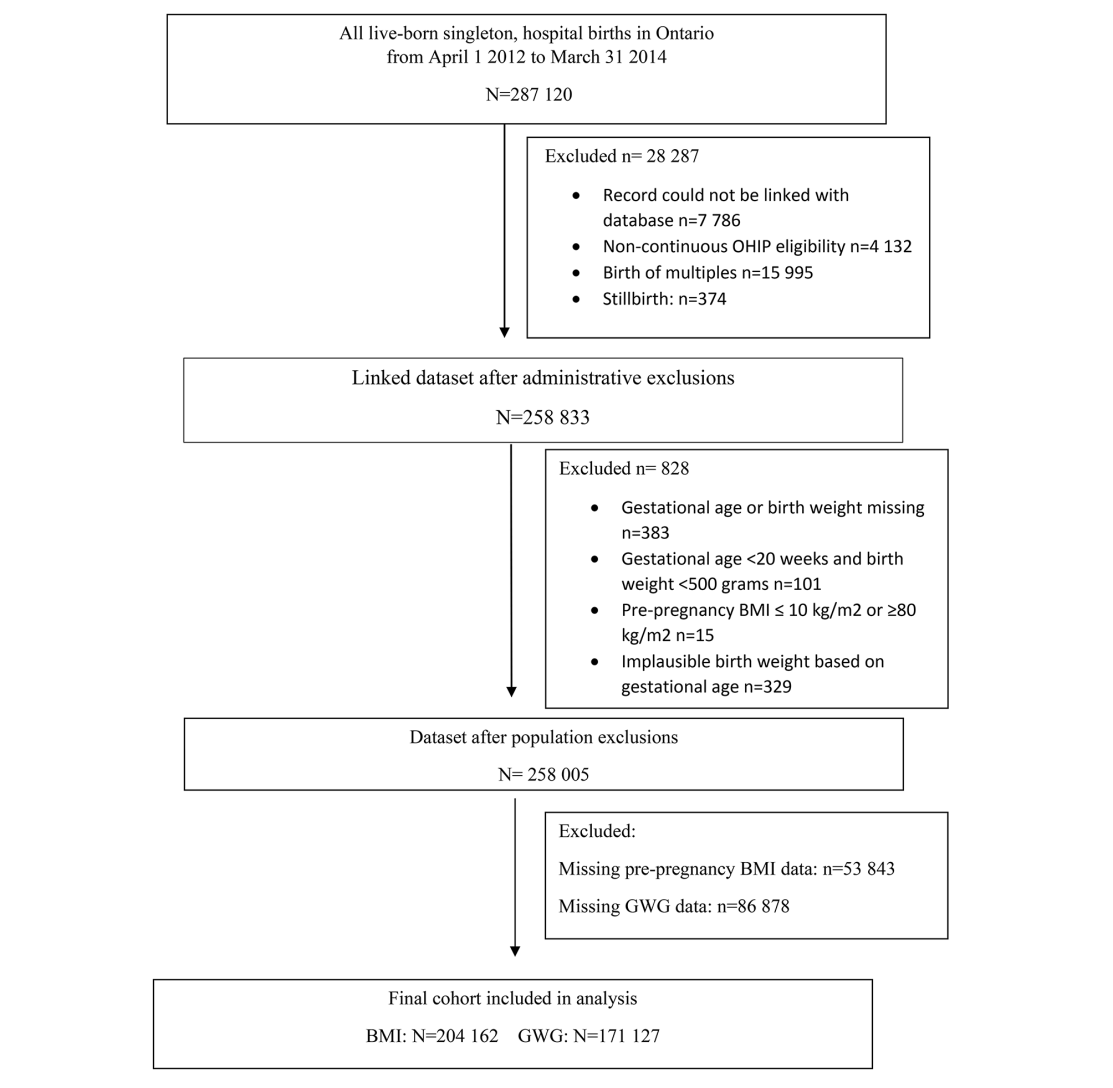
Study Flow Diagram

We identified 258,833 birth records in BORN from April 1, 2012 to March 31, 2014. Following exclusions (records that could not be linked within the databases, had non-continuous OHIP eligibility, multiple birth, stillbirth, missing data for gestational age or birth weight, gestation age less than 20 weeks or birth weight less than 500 g, pre-pregnancy BMI ≤ 10 kg/m2 or ≥ 80 kg/m2, or implausible birth weight based on gestational age) (Fig. 3), 258,005 infants were eligible for inclusion in the analysis. Of these records, there were 53,843 with missing BMI data. As only complete cases were analyzed in this study, 204,162 records were included in the analysis of maternal pre-pregnancy BMI. There were 86,878 records with missing GWG information. Therefore, 171,127 records were included in the GWG analysis.

Characteristics of the entire study population are presented in Table 1 and stratified by maternal pre-pregnancy BMI (Table S2A) and GWG (Table S2B). Overall mean maternal age was 30.5 years (SD: 5.41). The mean gestational age at birth was 38.9 weeks (SD: 1.81) and mean birth weight was 3362.3 g (SD: 547.9). As described above, a high proportion of records had missing data for pre-pregnancy BMI (20.9%) and GWG (33.7%). We reviewed missing data patterns prior to analyses to compare characteristics of records with and without complete maternal BMI data. An absolute standardized difference of > 10% was considered indicative of a different distribution between the two groups to inform whether data could be considered missing at random (Table S3). There were few differences in perinatal outcomes in those with complete versus missing weight information. Among those with complete information, the median pre-pregnancy BMI was 23.9 kg/m^2^ (mean: 25.5 kg/m^2^, SD: 6.42) and 21% had GWG in the recommended range.

**Table 1:** Maternal and infant characteristics of the study population

Characteristics	N	%
**All births**	258,005	100
**Maternal age (years)**		
<20	7012	2.7
20–24	29 983	11.6
25–29	71,571	27.7
30–34	92,040	35.7
35–40	50,852	19.7
≥41	6528	2.5
Missing	19	0
**Smoking during pregnancy**		
No	229,007	88.7
Yes	21,326	8.3
Missing	7672	3.0
**Neighbourhood income quintile**		
1 (lowest)	55,701	21.6
2	51,333	19.9
3	51,866	20.1
4	54,961	21.3
5 (highest)	41,856	16.2
Missing	2288	0.9
**Pre-pregnancy BMI (kg/m** ^**2**^ **)**		
<18.5 (underweight)	10,901	4.2
18.5–24.9 (normal)	108,170	41.9
25-29.9 (overweight)	48,141	18.7
≥30 (obese)	36,950	14.3
Missing	53,843	20.9
**Maternal weight gain during pregnancy**		
Below recommended	42,826	16.6
Recommended	54,302	21
Above recommended	73,999	28.7
Missing	86,878	33.7
**Infant sex**		
Male	132,231	51.3
Female	125,629	48.7
Missing	145	0.0
**Gestational age at birth (weeks)**		
<37	15,211	5.9
≥37	242,794	94.1
**Birth weight (grams)**		
<2500	11,762	4.6
≥2500	246,243	95.4
**SGA**		
No	234,120	90.7
Yes	23,653	9.2
Missing	232	0.09

### Mediation analysis results for pre-pregnancy BMI

Figure 2a illustrates the model construction for each step of the Baron-Kenny method and causal mediation for maternal pre-pregnancy BMI.

#### Step 1 (relationship between exposure and outcome)

The results suggested an increased rate of hospitalizations and ED visits for children born to overweight and obese persons compared to those born to normal weight persons. However, none of the other associations between pre-pregnancy BMI and paediatric health service use were strong enough to meet criteria for continuation to step 2 (Table S4).

#### Step 2 (relationship between exposure and mediator)

The results suggested that children born to mothers who were overweight or obese prior to pregnancy had lower rates of SGA (Table S5) relative to children born to normal weight persons. PTB demonstrated a nonsignificant positive relationship with pre-pregnancy BMI in the overweight and obese categories. As the magnitude of these IRRs were smaller than the range previously specified for inclusion, PTB was removed from subsequent mediation analyses related to maternal pre-pregnancy weight.

#### Step 3 (relationship between the mediator and outcome)

Results suggested that children born SGA had an increased rate of hospitalizations and a small decrease in the rate of ED visits compared to children who were not born SGA (Table S6).

Steps 1–3 in the adjusted analyses had thus suggested that SGA may partially explain the relationship between maternal weight and paediatric hospitalization. However, the decreased rate of SGA among children born to mothers who were overweight or obese prior to pregnancy (*step 2*) limited the potential for a causal mediation interpretation given that the direction of association was opposite to what would be expected for a causal mediator.

#### Step 4 (relationship of the exposure and mediators to the outcome)

Results indicated thatchildren born to persons who were overweight or obese still had increased rates of hospitalization compared to children born to mothers with normal pre-pregnancy BMI (Table S7), with effect estimates that were comparable to the Step 1 findings.

*Causal mediation analyses*:

Casual mediation analyses were implemented to assess the role of SGA in the association between maternal pre-pregnancy BMI and hospitalizations (Table 2) did not demonstrate any mediation by SGA: the percent of the effect mediated through SGA was minimal and most of the association was due to the direct effect of maternal weight on paediatric hospitalizations.

**Table 2:** Relationship between maternal pre-pregnancy BMI and infant health service utilization in the first 24 months of life mediated by SGA.

	SGA [Beta Estimate (95% CI)]						% Mediated	
	Total Effect		Direct Effect		Indirect Effect			
	Unadjusted	Adjusted*	Unadjusted	Adjusted*	Unadjusted	Adjusted*	Unadjusted	Adjusted*
*Hospitalizations*								
Overweight	0.02 (0.01, 0.03)	0.02 (0.01, 0.03)	0.02 (0.02, 0.03)	0.02 (0.01, 0.03)	0.00 (0.00–0.00)	0.00 (0.00–0.00)	**	**
Obese	0.05 (0.04, 0.06)	0.05 (0.04, 0.05)	0.05 (0.04, 0.06)	0.05 (0.04, 0.06)	0.00 (0.00–0.00)	0.00 (0.00–0.00)	**	**

* Adjusted for maternal age, maternal smoking status, infant sex, maternal pre-existing medical conditions (diabetes, hypertension) and neighbourhood income quintile

**The resulting percent mediated is not theoretically possible and has been omitted from the results

Referent group is children born to mothers with normal pre-pregnancy BMI

### Mediation analysis results for GWG

Figure 2b illustrates the model construction for each step of the Baron-Kenny method and causal mediation for gestational weight gain.

*Step 1 (relationship between exposure and outcome)*:

Results suggested an increased rate of hospitalizations for children born to persons with below recommended GWG. Children born to mothers with above recommended GWG had an increased rate of ED visits compared to those born to mothers with recommended GWG. However, all other associations were small or nonsignificant and removed from all subsequent analyses (Table S8).

#### Step 2 (relationship between exposure and mediator)

Results suggested that children born to mothers with below recommended GWG had an increased rate of PTB and SGA, compared to children born to mothers with recommended GWG. Children born to mothers with above recommended GWG were not measurable in this model due to a Hessian error, likely attributed to low cell sizes and lack of variation within the matrix. The model for above recommended GWG was unable to achieve an estimate after adjustment for number of iterations and was thus omitted from subsequent analyses (Table S9).

#### Step 3 (relationship between the mediator and outcome)

Children born preterm and those with SGA had increased rates of hospitalizations (Table S10).

As a result of Steps 1–3 in the adjusted analyses, it was suggestive that PTB and SGA may be mediators in the relationship between below recommended GWG and infant hospitalizations.

#### Step 4 (relationship of the exposure and mediators to the outcome)

The association of below recommended GWG with increased hospitalizations was diminished and non-significant after controlling for PTB and was diminished after controlling for SGA (Table S11).

*Casual Mediation Analysis*:

Causal mediation analyses were completed to assess the roles of SGA and PTB in the association between below recommended GWG and infant hospitalizations (Table 3). As shown in Table 3, PTB appeared to be responsible for a majority of the association between below recommended GWG and infant hospitalizations, as 56.7% of the small association was mediated by PTB. SGA only mediated 6.8% of the relationship between below recommended GWG and hospitalizations (Table 3). However, given the small magnitude of all the associations, the percentages mediated should be interpreted with caution.

**Table 3:** Relationship between maternal gestational weight gain and infant hospitalizations in the first 24 months of life- mediated by PTB or SGA

	[Beta Estimate (95% CI)]						% Mediated	
	Total Effect		Direct Effect		Indirect Effect			
	Unadjusted	Adjusted*	Unadjusted	Adjusted*	Unadjusted	Adjusted*	Unadjusted	Adjusted*
*Hospitalizations, mediated by PTB*								
Below recommended	0.02 (0.01, 0.03)	0.02 (0.01, 0.03)	0.01 (0.00, 0.02)	0.01 (0.00, 0.02)	0.01 (0.01, 0.01)	0.01 (0.01, 0.01)	54.6	56.7
*Hospitalizations, mediated by SGA*								
Below recommended	0.02 (0.01, 0.03)	0.02 (0.01, 0.03)	0.02 (0.01, 0.03)	0.02 (0.01, 0.03)	0.00 (0.00, 0.00)	0.00 (0.00, 0.00)	7.3	6.8

* Adjusted for maternal age, maternal smoking status, infant sex, maternal pre-existing medical conditions (diabetes, hypertension), and neighbourhood income quintile

**The resulting percent mediated is not theoretically possible and has been omitted from the results

Referent group is children born to mothers with recommended GWG

## Discussion

The results of the study suggested that PTB and SGA (to a small degree) may be contributing factors to increased hospitalizations in children born to mothers with below recommended GWG. However, the magnitude of association between maternal weight and paediatric health service utilization was relatively small, impacting the ability to estimate the indirect effect of maternal weight through adverse birth outcomes.

### Main findings

The results of this study regarding pre-pregnancy BMI are suggestive of inconsistent mediation. When a relationship within the causal pathway has a different direction of association compared to the other effects, it can result in suppression of the total effect [21]. Within this study, increased maternal pre-pregnancy BMI was associated with increased health service utilization. However, increased pre-pregnancy BMI was associated with decreased rates of SGA. Since the directionality of the effects opposite, the resulting suppression of the total effect limits the interpretation of the findings.

PTB and to a lesser extend SGA appeared to partially explain the observed higher rates of child hospitalizations in the first 24 months of life among children born to mothers with below recommended maternal GWG, compared to children born to mothers with recommended GWG, but was not associated with pre-pregnancy BMI. PTB and SGA are associated with longstanding health implications [22]. The results of these analyses suggest that PTB and SGA may lie on the causal pathway and contribute to the increase in hospitalizations of children born to persons with below recommended GWG within the first twenty-four months following birth.

### Interpretation

Our findings are consistent with other studies in that children born to overweight or obese mothers have increased health service utilization [23]. Children born to obese mothers [24], and those with above recommended GWG [25] are at increased risk of obesity themselves, as well as associated disease states including diabetes and cardiovascular disease [26]. They are also at increased risk of other adverse conditions, such as developmental delay [27] and impaired neurodevelopment extending into childhood [28]. Research suggests that normal pre-pregnancy BMI and recommended GWG are associated with a decreased risk of PTB [29], thereby limiting the potential consequences on long-term outcomes [30]. This study is novel as it quantifies the relationship of maternal weight to several aspects of pediatric health service utilization. Further, it addresses the relationship to gestational weight gain, which has not previously been investigated.

### Strengths and limitations

A strength of this study is availability of maternal weight and height information, linked with paediatric health service utilization in a large population, given the scarcity of maternal weight and height in electronic databases. Population-based information from a birth registry augments the generalizability of the findings to other populations with similar characteristics. A key limitation was the high proportion of missing data on maternal pre-pregnancy BMI and GWG. Self reported pre-pregnancy weight should be interpreted with caution given the potential for reporting bias. Additionally, there may be other factors not explored that may explain the relationship between maternal weight and health service utilization, including the impact of maternal metabolic disease, beyond maternal diabetes or hypertension, on the child during in utero development. Finally, GWG is impacted by gestational length, which can be shortened by PTB. Despite the small proportion of preterm births within the cohort, this is an important limitation within this study, given that it may inflate the perceived role of PTB as a mediator in the relationship of maternal GWG to paediatric hospitalizations. Additionally, other biological mechanisms not captured in this study may lead to PTB, and thereby contribute to future health service utilization.

## Conclusion

In conclusion, PTB may be a contributor to the increased hospitalizations in children born to mothers with below recommended GWG. Increased paediatric health service utilization in children born to mothers who were overweight or living with obesity prior to pregnancy does not appear to be related to SGA or PTB, suggesting that this observed association is due to either maternal weight status alone or due to other unmeasured mediators. These findings are important as they indicate that adverse birth outcomes may mediate the relationship between maternal weight and pediatric health service utilization and warrants further investigation.

## Electronic supplementary material

Below is the link to the electronic supplementary material.

Supplementary Material 1

## Data Availability

The dataset from this study is held securely in coded form at ICES. While legal data sharing agreements between ICES and data providers (e.g., healthcare organizations and government) prohibit ICES from making the dataset publicly available, access may be granted to those who meet pre-specified criteria for confidential access, available at www.ices.on.ca/DAS (email: das@ices.on.ca). The full dataset creation plan and underlying analytic code are available from the authors upon request, understanding that the computer programs may rely upon coding templates or macros that are unique to ICES and are therefore either inaccessible or may require modification. For additional follow up please contact the corresponding author.

## Abbreviations

aIRR: Adjusted incidence rate ratio
AOR: Adjusted Odds Ratio
ARR: Adjusted Relative Risk
BMI: Body Mass Index
CI: Confidence interval
CIHI: Canadian Institute for Health Information
DAD: Discharge Abstract Database
ED: Emergency Department
GWG: Gestational Weight Gain
IKN: ICES Key Number
IRR: Unadjusted incidence rate ratios
kg: Kilograms
lb: Pounds
m: Metres
MI: Multiple Imputation
NACRS: National Ambulatory Care Reporting System
NICU: Neonatal Intensive Care Unit
NS: Not significant
OR: Odds Ratio
OHIP: Ontario Health Insurance Plan
PTB: Preterm birth
RPDB: Registered Persons Database
SD: Standard deviation
SGA: Small for gestational age
WHO: World Health Organization
