# Supplementary material for: Maternal weight and paediatric health use: mediating role of adverse birth outcomes: a retrospective cohort study
Source: BMC Pregnancy Childbirth. 2023 Jul 31;23:546. doi: 10.1186/s12884-023-05744-w (PMC10388559; doi:10.1186/s12884-023-05744-w)
Supplement: Supplementary file 1 — Supplementary Material 1 [file 12884_2023_5744_MOESM1_ESM.pdf]

Supplemental Information

Table S1: Health Canada gestational weight gain recommendations [11].

Pre-Pregnancy BMI	Recommended Total Weight Gain	
	Kg	lb
< 18.5 kg/m^2	12.5 - 18	28 - 40
18.5-24.9 kg/m^2	11.5 - 16	25 - 35
25.0-29.9 kg/m^2	7 - 11.5	15 - 25
≥30.0 kg/m^2	5 - 9	11 - 20

Table S2A*:* Characteristics stratified by pre-pregnancy BMI

**Characteristics**	**Total**	**Under-weight (N)**	**Normal weight (N)**	**Over-weight (N)**	**Obese (N)**	**Missing (N)**
**All births**	258005	10901	108170	48141	36950	53843
**Maternal age (years)**						
<20	7012	767	3233	946	682	1384
20-24	29983	2023	12445	5292	4428	5795
25-29	71571	3317	30150	13358	10686	14060
30-34	92040	3186	39428	17384	12670	19372
35-40	50852	1475	20509	9871	7478	11519
≥41	6528	132	2403	1289	1004	1700
Missing	19	**	**	**	**	13
**Smoking during pregnancy**						
No	229007	9226	98264	43561	32529	45427
Yes	21326	1466	8195	3889	3901	3875
Missing	7672	209	1711	691	520	4541
**Neighbourhood income quintile**						
1 (lowest)	55701	2672	21064	10695	9189	12081
2	51333	2371	20797	9679	7780	10706
3	51866	2072	21846	9857	7550	10541
4	54961	2095	23982	10136	7274	11474
5 (highest)	41856	1596	19607	7345	4758	8550
Missing	2288	95	874	429	399	491
**Infant sex**						
Male	132231	5623	55459	24631	18939	27579
Female	125629	5274	52644	23477	17990	2624
Missing	145	**	67	33	21	20
**Gestational age at birth (weeks)**						
<37	15211	715	5670	2697	2244	3885
≥37	242794	10186	102500	45444	34706	49958
**Birth weight (grams)**						
<2500	11762	807	4648	1823	1416	3068
≥2500	246243	10094	103522	46318	35534	50775

** Compressed due to small cell size

Table S2B: Characteristics stratified by GWG

Characteristics	Total	Below recommend. (N)	Recommend. (N)	Above recommend. (N)	Missing (N)
**All births**	258005	75861	54302	73999	53843
**Maternal age (years)**					
<20	7012	1900	1276	2452	1384
20-24	29983	8312	6034	9842	5795
25-29	71571	20017	15661	21833	14060
30-34	92040	27328	19956	25384	19372
35-40	50852	16079	10186	13068	11519
≥41	6528	2221	1189	1418	1700
Missing	19	**	**	**	13
**Smoking during pregnancy**					
No	229007	67911	49420	66249	45427
Yes	21326	6206	4262	6983	3875
Missing	7672	1744	620	767	4541
**Neighbourhood income quintile**					
1 (lowest)	55701	16647	10981	15992	12081
2	51333	15192	10689	14746	10706
3	51866	15021	11000	15304	10541
4	54961	15468	12078	15941	11474
5 (highest)	41856	12875	9110	11321	8550
Missing	2288	658	444	695	491
**Infant sex**					
Male	132231	38127	27758	38767	27579
Female	125629	37689	26516	35180	26244
Missing	145	45	28	52	20
**Gestational age at birth (weeks)**					
<37	15211	5641	2720	2965	3885
≥37	242794	70220	51582	71034	49958
**Birth weight (grams)**					
<2500	11762	4914	1995	1785	3068
≥2500	246243	70947	52307	72214	50775

** Compressed due to small cell size

Table S3: Sensitivity Analyses of Factors by Missing BMI and GWG Data

**Variable**		**BMI Standardized Difference***	**GWG Standardized Difference***
**Maternal smoking**	Non-Smoker	**-0.17**	-0.09
	Smoker	-0.05	-0.10
	Missing	**0.54**	**0.48**
**Gender**	Male	0.00	-0.01
	Female	0.00	0.01
	missing	0.03	0.00
**LGA (≥90th percentile)**	No	0.04	0.04
	Yes	-0.04	-0.04
	missing	-0.01	0.00
**SGA ≤10th percentile)**	No	**-0.28**	-0.04
	Yes	0.02	0.04
	missing	**0.45**	0.04
**Presentation**	Breech	-0.02	0.01
	Cephalic	**-0.57**	**-0.45**
	Transverse	0.00	0.01
	missing	**0.65**	**0.65**
**Maternal Age (years)**	<20	-0.01	-0.05
	20-24	-0.03	-0.09
	25-29	-0.05	-0.08
	30-34	0.01	0.04
	35-40	0.05	**0.10**
	≥41	0.05	0.07
	missing	0.09	0.08
**Birth weight (grams)**	<2500	0.07	0.08
	≥2500	-0.07	-0.08
**Gestational Age (weeks)**	<37	0.07	0.08
	≥37	-0.07	-0.08
**Time to Death**	≤ 42	0.00	0.03

	42-365	0.00	0.01
	365-730	0.00	0.01
	missing	0.00	-0.02
**Birth Year**	2012	**0.14**	**0.10**
	2013	-0.09	-0.05
	2014	-0.07	-0.08

*Standardized differences were calculated to compare the absolute differences between those with complete maternal weight (pre-pregnancy BMI or GWG) compared to those with missing maternal weight data to ascertain if there were differences in population characteristics among those with and those without weight data. An absolute difference of >10% was considered indicative of a different distribution between the two groups.

Pre-Pregnancy BMI: Baron-Kenny Results

Table S4: Relationship between maternal pre-pregnancy BMI and rate of infant hospitalizations, physician visits and ED visits in the first 24 months of life (Step 1 of Baron-Kenny, Relationship A in Figure 1)

	**Hospitalizations: IRR (95% CI)**		**Physician Visits: IRR (95% CI)**		**ED Visits: IRR (95% CI)**	
	Unadjusted	Adjusted*	Unadjusted	Adjusted*	Unadjusted	Adjusted*
Underweight	1.00 (0.94,1.05)	0.96 (0.91,1.02)	1.01 (1.00, 1.02)	1.02 (1.01,1.03)	1.06 (1.03,1.09)	0.96 (0.93,0.98)
Normal weight (ref)	1.0	1.0	1.0	1.0	1.0	1.0
Overweight	1.10 (1.07,1.14)	1.10 (1.07,1.13)	1.03 (1.05, 1.06)	1.03 (1.02,1.04)	1.12 (1.10,1.14)	1.13 (1.11,1.15)
Obese	1.25 (1.21,1.29)	1.23 (1.19,1.27)	1.06 (1.05, 1.06)	1.05 (1.04,1.06)	1.31 (1.29,1.33)	1.30 (1.28,1.32)

* Adjusted for maternal age, maternal smoking status, infant sex, maternal pre-existing medical conditions (diabetes, hypertension), and neighbourhood income quintile.

Table S5: Relationship between maternal pre-pregnancy BMI and adverse event in the first 24 months of life (Step 2 of Baron-Kenny, Relationship B in Figure 1)

	**PTB: IRR (95% CI)**		**SGA: IRR (95% CI)**	
	Unadjusted	Adjusted*	Unadjusted	Adjusted*
Normal weight (ref)	1.0	1.0	1.0	1.0
Overweight	1.07 (1.02, 1.12)	1.04 (0.99,1.09)	**	0.73 (0.70,0.76)
Obese	1.16 (1.10, 1.22)	1.07 (1.02,1.13)	**	0.63 (0.60 0.66)

* Adjusted for maternal age, maternal smoking status, infant sex, maternal pre-existing medical conditions (diabetes, hypertension), and neighbourhood income quintile.
** Hessian error message, attributed to the low cell sizes and lack of variation within the matrix.

Table S6: Relationship between SGA and rate of infant hospitalizations and ED visits in the first 24 months of life (Step 3 of Baron-Kenny, Relationship C in Figure 1)

	**Hospitalizations: IRR (95% CI)**		**ED Visits: IRR (95% CI)**	
	Unadjusted	Adjusted*	Unadjusted	Adjusted*
SGA	1.28 (1.24,1.33)	1.26 (1.21, 1.31)	1.01 (0.99, 1.03)	0.97 (0.95, 0.99)

* Adjusted for maternal age, maternal smoking status, infant sex, maternal pre-existing medical conditions (diabetes, hypertension), and neighbourhood income quintile.

Table S7: Relationship between maternal pre-pregnancy BMI and and rate of infant hospitalizations and ED visits in the first 24 months of life after controlling for SGA (step 4 of Baron-Kenny mediation)

	**Hospitalizations: IRR (95% CI)**	
	Unadjusted	Adjusted*
Normal weight (ref)	1.0	1.0
Overweight	1.11<br>(1.08, 1.15)	1.11<br>(1.08, 1.14)
Obese	1.26<br>(1.22,1.30)	1.24<br>(1.20, 1.28)

* Adjusted for maternal age, maternal smoking status, infant sex, maternal pre-existing medical conditions (diabetes, hypertension) and neighbourhood income quintile.

Gestational Weight Gain: Baron-Kenny Results

Table S8: Relationship between maternal gestational weight gain and rate of infant hospitalizations, physician visits and ED visits in the first 24 months of life (Step 1 of Baron Kenny, Relationship A in Figure 1)

	**Hospitalizations: IRR (95% CI)**		**Physician Visits: IRR (95% CI)**		**ED Visits: IRR (95% CI)**	
	Unadjusted	Adjusted*	Unadjusted	Adjusted*	Unadjusted	Adjusted*
Below recommended	1.11 (1.07,1.14)	1.10 (1.06,1.14)	1.05 (1.04,1.06)	1.05 (1.04,1.06)	1.02 (1.00,1.04)	1.00 (0.98,1.01)
Recommended (ref)	1.0	1.0	1.0	1.0	1.0	1.0
Above recommended	1.06 (1.03,1.09)	1.04 (1.01,1.08)	1.01 (1.01,1.02)	1.01 (1.01,1.02)	1.13 (1.12,1.15)	1.10 (1.09,1.12)

* Adjusted for maternal age, maternal smoking status, infant sex, maternal pre-existing medical conditions (diabetes, hypertension), and neighbourhood income quintile.

Table S9: Relationship between maternal gestational weight gain and adverse event in the first 24 months of life (Step 2 of Baron Kenny, Relationship B in Figure 1)

	**PTB: IRR (95% CI)**		**SGA: IRR (95% CI)**	
	Unadjusted	Adjusted*	Unadjusted	Adjusted*
Below recommended	1.78 (1.71,1.86)	1.77 (1.69,1.84)	1.84 (1.78,1.91)	1.82 (1.76,1.88)
Recommended (ref)	1.0	1.0	1.0	1.0
Above recommended	**	**	**	**

* Adjusted for maternal age, maternal smoking status, infant sex, maternal pre-existing medical conditions (diabetes, hypertension), and neighbourhood income quintile.
** Hessian error message, attributed to the low cell sizes and lack of variation within the matrix.

Table S10: Relationship between adverse event and rate of infant hospitalizations in the first 24 months of life (Step 3 of Baron Kenny, Relationship C in Figure 1)

	**Hospitalizations: IRR (95% CI)**	
	Unadjusted	Adjusted*
PTB	3.10<br>(2.99,3.21)	3.01<br>(2.90,3.12)
SGA	1.28<br>(1.24, 1.33)	1.26<br>(1.22, 1.31)

* Adjusted for maternal age, maternal smoking status, infant sex, maternal pre-existing medical conditions (diabetes, hypertension), and neighbourhood income quintile.

Table S11: Relationship between GWG and and rate of infant hospitalizations in the first 24 months of life after controlling for PTB and SGA (step 4 of Baron Kenny mediation)

	**PTB: IRR (95% CI)**		**SGA: IRR (95% CI)**	
	Unadjusted	Adjusted*	Unadjusted	Adjusted*
Below recommended	1.00<br>(0.97,1.03)	1.01<br>(0.98,1.04)	1.05<br>(1.02, 1.09)	1.06<br>(1.02, 1.09)
Recommended (ref)	1.0	1.0	1.0	1.0

* Adjusted for maternal age, maternal smoking status, infant sex, maternal pre-existing medical conditions (diabetes, hypertension), and neighbourhood income quintile.
